# Georeferenced Scanning System to Estimate the Leaf Wall Area in Tree Crops

**DOI:** 10.3390/s150408382

**Published:** 2015-04-10

**Authors:** Ignacio del-Moral-Martínez, Jaume Arnó, Alexandre Escolà, Ricardo Sanz, Joan Masip-Vilalta, Joaquim Company-Messa, Joan R. Rosell-Polo

**Affiliations:** Research Group in AgroICT & Precision Agriculture, Department of Agricultural and Forest Engineering, University of Lleida. Av. Rovira Roure, 191, Lleida 25198, Spain;E-Mails: jarno@eagrof.udl.cat (J.A.); aescola@eagrof.udl.cat (A.E.); rsanz@eagrof.udl.cat (R.S.); jmv@eagrof.udl.cat (J.M.-V.); jcompany@alumnes.udl.cat (J.C.-M.); jr.rosell@eagrof.udl.cat (J.R.R.-P.)

**Keywords:** pixelated leaf wall area (PLWA), LiDAR, RTK-GPS georeferencing, inertial sensor, tree crops

## Abstract

This paper presents the use of a terrestrial light detection and ranging (LiDAR) system to scan the vegetation of tree crops to estimate the so-called pixelated leaf wall area (PLWA). Scanning rows laterally and considering only the half-canopy vegetation to the line of the trunks, PLWA refers to the vertical projected area without gaps detected by LiDAR. As defined, PLWA may be different depending on the side from which the LiDAR is applied. The system is completed by a real-time kinematic global positioning system (RTK-GPS) sensor and an inertial measurement unit (IMU) sensor for positioning. At the end, a total leaf wall area (LWA) is computed and assigned to the X, Y position of each vertical scan. The final value of the area depends on the distance between two consecutive scans (or horizontal resolution), as well as the number of intercepted points within each scan, since PLWA is only computed when the laser beam detects vegetation. To verify system performance, tests were conducted related to the georeferencing task and synchronization problems between GPS time and central processing unit (CPU) time. Despite this, the overall accuracy of the system is generally acceptable. The Leaf Area Index (LAI) can then be estimated using PLWA as an explanatory variable in appropriate linear regression models.

## 1. Introduction

Precision agriculture (PA) can be defined as the practice of managing agronomic systems that takes into consideration the field variability. PA relies on technologies, such as proximal and remote sensors, global navigation satellite systems (GNSS), decision support systems and variable-rate agricultural machinery.

Light detection and ranging (LiDAR) has been used in agriculture and forestry in three main areas. First, it has been used to differentiate between areas with and without vegetation, as in the case of [1]. In [2], a mixed system, including a LiDAR sensor and hyperspectral image sensor mounted on a ground-based vehicle, was used to distinguish between conifers and deciduous varieties in a population of 168 trees. Second, LiDAR has been used in machinery guidance systems [3]. Third, LiDAR sensors have been used to inventory vegetation [4–7] and biomass [8].

In the past two decades, GNSS have been widely applied to agriculture; for example, RTK-GPS has found wide application in steering systems [9–18]. In contrast, the use of RTK-GPS to georeference agricultural parameters has been notably less widespread [19–21]. Some other applications of RTK-GPS are the creation of digital terrain models and the positioning of elements.

The use of several sensors with complementary data requires a system capable of combining the information supplied by different devices. One way is to establish a continuous time frame, so that each event is labeled with the time at which it takes place (timestamp). A timestamp is used to relate and connect the sensor measurements by interpolation. According to the literature, there are three ways of applying this timestamp:

1. Using a timeboard (hardware) that gives the time at which each sentence of data reaches the timeboard [22].
2. Sending one pulse per second (pps) from the GPS to the other sensors [8,23]. The work in [24] has evaluated the precision of synchronization using this pps procedure.
3. Giving the CPU time when data enter the computer [21]. This is the weakest solution, because it depends on the precision of the CPU time and on the delay between the time of the measurement and the time of data transmission. This is, however, the cheapest alternative.

In working with GPS time in the first and second methods listed above, it is necessary to send a pps to synchronize the CPU. If the first alternative is selected, it is also necessary to use new hardware. It should be noted that some GPS equipment cannot send this pps pulse. For example, the 1200 (Leica Geosystems AG, Heerbrugg, Switzerland) RTK-GPS would need new hardware in order to produce this pulse.

Estimating LWA is of interest in agriculture, particularly when pesticides are applied to fruit orchards, and dose rates must be adapted according to the characteristics of the vegetation. Moreover, in Europe, the use of the LWA approach is recommended to harmonize dose expressions. So far, different parameters have been used in each country. LWA can be expressed as ([25]).

(1) $$\begin{array}{c} \text{LWA}=2\cdot \left(\text{cancopy height}/\text{row distance}\right)\cdot \text{ground area}=\\ =2\cdot \left(\text{cancopy height}\right)\cdot \text{number of rows}\cdot \text{lenght of rows} \end{array}$$

where the value of two in Equation (1) is used in order to take into consideration both sides of the vegetation wall. This parameter has the following advantages: (i) it is linked to the vegetation dimension; (ii) it can be related to other ways of establishing the dose, e.g., as the volume per ha; and (iii) there is a linear relation between the LWA and the deposition of plant protection products (PPP) onto the leaves [25].

The aim of this work is to assess a terrestrial LiDAR system for estimating LWA in a more accurate way, considering the canopy gaps on either side of tree crops. The novelty of the proposed system is that it introduces the concept of PLWA, in which a vertical projected area (or pixel) is assigned to each intercepted point at the canopy level to finally estimate the leaf wall area by adding up the individual pixels. The on-the-go estimation of this parameter allows varying doses of PPP, according to the spatial variability of PLWA within the orchard.

## 2. Material and Methods

The proposed terrestrial LiDAR system is made up of a synchronization subsystem, a crop canopy data acquisition subsystem and a post-processing subsystem.

### 2.1. Subsystem to Synchronize the Data

**Figure 1:**
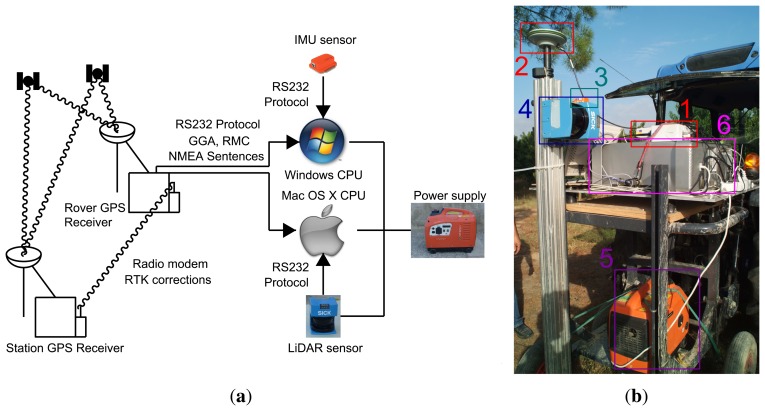
Terrestrial LiDAR system in combination with the IMU and RTK-GPS receiver. (**a**) Connection diagram; (**b**) system components: 1, RTK-GPS receiver; 2, GPS antenna; 3, IMU; 4, LiDAR; 5, power supply; 6, Mac OS X computer.

The system presented here required two computers working together (Figure 1). The first computer operated Windows^©^ (Microsoft, Redmond, WA, USA) and received data from the IMU and RTK-GPS, synchronizing the CPU and GPS times. The second computer operated Mac OS X Snow Leopard^©^ (Apple, Cupertino, CA, USA) and received data from LiDAR and RTK-GPS to synchronize the CPU time and obtain GPS coordinates. Both computers acquired data from the same RTK-GPS receiver. In this way, it was possible to relate data from the two computers. The scanning system was initially designed to use only the second computer. We adopted this scheme because the Mac OS offers greater accuracy in the administration of time. However, only Windows drivers were available to connect the IMU sensor to a computer. For that reason, the final system used a Mac OS computer and a Windows computer.

### 2.2. Subsystem to Acquire Crop Canopy Data

In this system, CPU time was used to timestamp data from the different components. Nevertheless, a computation of the delay between the measurement and the timestamp with the CPU was necessary to analyze the accuracy and stability of the timestamp. However, this is not possible for all sensors. Only in the case of GPS, since GPS National Marine Electronics Association (NMEA) sentences provide us with the time when the measurement took place, can it be measured. In any case, this value can be considered as the mean delay for the other sensors, because they use the same type of communication (serial communication).

Data sentences and their corresponding timestamps were saved in files (one file per sensor). The timestamp was saved in the file just before the corresponding sentence and had the following structure: $*GPTIM, 734697.40780671*. This expresses the date and time, in days and fractions of a day, following the MATLAB^©^ serial date number (MathWorks, MA, USA).

**Figure 2:**
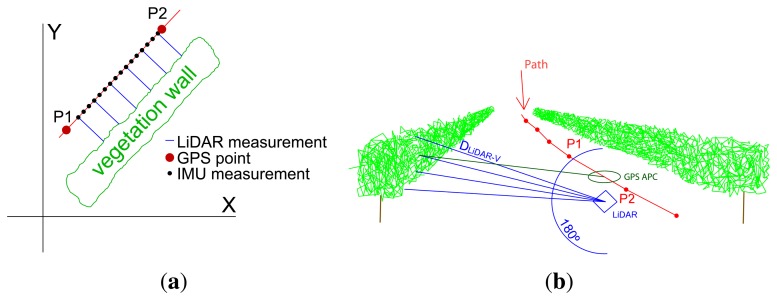
Scheme of sensor measurements in an orchard. The GPS antenna phase center (dark green lines) and LiDAR (blue lines) are separated by a distance. As GPS provides global coordinates of the measurements, the canopy point cloud must be referred to the GPS antenna phase center. (**a**) Measurements in 2D; (**b**) measurements in 3D.

The LiDAR sensor used was an LMS-200 model (SICK AG, Waldkirch, Germany) based on the time-of-flight principle. The communication protocol was RS232, working at a baud rate of 19,200. The scanning range or field of view was 180° (Figure 2b), with an angular resolution of one degree (Figure 2b). There were two reasons for using this configuration: (i) it was very important to use the height of the ground (nadir point, 180° measurement) as a reference to detect points on the ground. The LiDAR configuration allowed two options: 0–100° or 0–180°. The 0–180° option was chosen, since 0–100° was not enough. However, the first measurements of the scan were loosened, because they were oriented toward the sky; (ii) The angular resolution affects the number of scans per second. The higher the resolution, the longer the distance between scans. Therefore, the distance between scans is shorter with 1° than with 0.25°. The configuration of 1° and 1 m/s allows a distribution in which the distance between points of the scan and the distance between scans are similar. With this configuration, the LiDAR sensor performed around 16 scans per second, including 181 points per scan. Every LiDAR sentence begins with $GPLMS.

**Figure 3:**
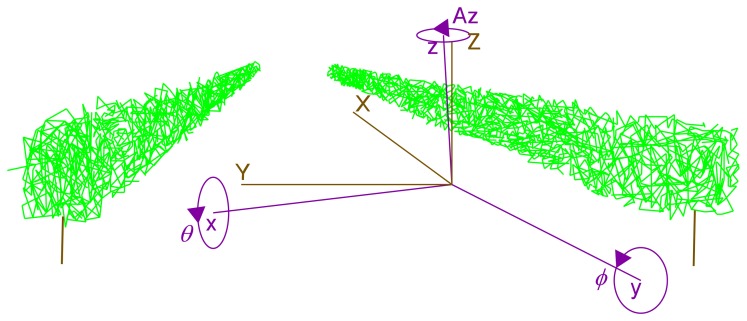
UTM coordinates (brown, capital letters) and system coordinates (dark violet, lower case letters). Rotations *Az, ϕ* and *θ* transform a vector from system orientation to absolute (UTM) orientation.

The IMU sensor used was the MTI model (Xsens Technologies, Enschede, the Netherlands), because of its small in size. It provided 3D orientation, calibrated 3D acceleration, a 3D rate of turn (gyro rate) and 3D Earth magnetic field data. The position where the IMU took measurements is represented by black dots in Figure 2a. In short, a 3 × 3 rotation matrix was applied to compensate for movements of the system during scanning (Equation (2)). This made it possible to rotate the system coordinates (vehicle system) to UTM coordinates (global system) (Figure 3):

(2) $$R=\left(\begin{array}{ccc} \cos\left(\theta \right)\cos\left(Az\right) & \sin\left(\phi \right)\sin\left(\theta \right)\cos\left(Az\right)-\cos\left(\phi \right)\sin\left(Az\right) & \cos\left(\phi \right)\sin\left(\theta \right)\cos\left(Az\right)+\sin\left(\phi \right)\sin\left(Az\right)\\ \cos\left(\theta \right)\sin\left(Az\right) & \sin\left(\phi \right)\sin\left(\theta \right)\sin\left(Az\right)+\cos\left(\phi \right)\cos\left(Az\right) & \cos\left(\phi \right)\sin\left(\theta \right)\sin\left(Az\right)-\sin\left(\phi \right)\cos\left(Az\right)\\ -\sin\left(\theta \right) & \sin\left(\phi \right)\cos\left(\theta \right) & \cos\left(\phi \right)\cos\left(\theta \right) \end{array}\right)$$

The rotation matrix is the first set of parameters to transform the coordinate system. The ( $\vec{\text{v}}+\vec{{\text{u}}_{\text{G}}}$) shown later will complete the transformation parameters.

The IMU sensor gave a measurement every 0.015 s. Every IMU sentence begins with *$GPINS*, e.g., *$GPTIM, 734697.40780671*

*$GPINS, 0.07265472,* −*0.98958379, 0.12427896, 0.99735457, 0.07180356,* − *0.01132034, 0.00227875, 0.12477267, 0.99218273*,

the last nine parameters being the values of the rotation matrix *R*, as can be seen in the following matrix:

$$\left(\begin{array}{ccc} 0.07265472 & -0.98958379 & 0.12427896\\ 0.99735457 & 0.07180356 & -0.01132034\\ 0.00227875 & 0.12477267 & 0.99218273 \end{array}\right)$$

As the azimuth calculated by the IMU has worse precision than the azimuth calculated with RTK-GPS, Equation (2) was calculated using *Az* from GPS and *θ, ϕ* from IMU.

Finally, an RTK-GPS receiver was chosen to georeference the acquired data. Information about the GPS was sent from the GPS receiver to the computer using National Marine Electronics Association (NMEA) 0183 sentences. The selected sentences were GGA (GPS fix data) because they have three coordinates for full positioning, and RMC (recommended minimum data for GPS), which provides the time and date. Specifically, the RTK-GPS used consisted of two 1200 Leica receivers, working at a baud rate of 9600, 2-Hz output NMEA data and radio-modem transmission of RTK corrections.

The three sensors used in the system must be geometrically related to one another. The system had an origin at the GPS antenna phase center (GPS APC) and had the axis oriented as follows:

- x-axis: oriented at 90°, parallel to the laser beam.
- y-axis: oriented in the forward direction.
- z-axis: oriented at 0°.

It is important to realize that the defined axes are not physical orientations, like the vertical (plumb line) or horizontal, since it is a coordinate system in movement. Although the x- and y-axes are defined using laser beam orientations, in order to simplify the task in the field, the orientations were taken using the LMS200 housing.

### 2.3. Subsystem (Algorithm) to Process the Acquired Data

The data processing followed three steps: (i) determination of the coordinates of the intercepted points between the LiDAR sensor and the canopy; (ii) filtering of no canopy points (omitted from computation); and (iii) estimation of the PLWA.

The first step was to calculate the UTM coordinates of the LiDAR points. Figure 2a shows measurements taken between two consecutive GPS points, *P*1 and *P*2. The absolute position of the GPS APC at the time when the LiDAR took measurements was interpolated between the two GPS points. The laser beam from the LiDAR sensor (blue lines in Figure 2a) was corrected using the IMU information (black dots in Figure 2a), according to the direction of motion, angle *ϕ* and perpendicular direction to the above angle, *θ*.

**Figure 4:**
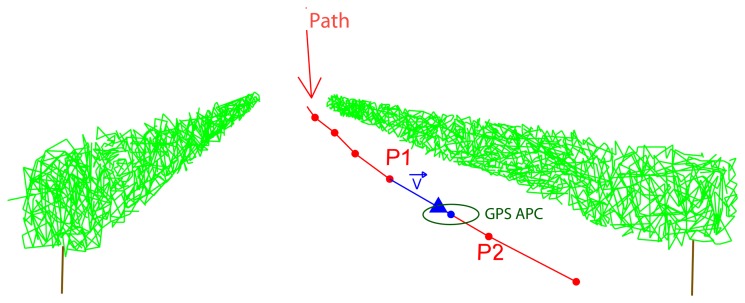
GPS antenna phase center (APC) position interpolated between P1 and P2 points.

Two main vectors were obtained in order to calculate the UTM coordinates of the LiDAR impacts. The first vector v⃗ goes from each GPS point to the GPS APC (Figure 4).

(3) $$\vec{\text{v}}=\left(\left(\begin{array}{c} X_{P2}\\ Y_{P2}\\ Z_{P2} \end{array}\right)-\left(\begin{array}{c} X_{P1}\\ Y_{P1}\\ Z_{P1} \end{array}\right)\right)\cdot \frac{t_{L}-t_{P1}}{t_{P2}-t_{P1}}$$

where: $\left(\begin{array}{c} X_{P1}\\ Y_{P1}\\ Z_{P1} \end{array}\right)$are the GPS coordinates at point *P*1, $\left(\begin{array}{c} X_{P2}\\ Y_{P2}\\ Z_{P2} \end{array}\right)$ are the GPS coordinates at point *P*2, *t_L_* is the timestamp of a specific LiDAR scan, *t_P_*_1_ is the timestamp of the NMEA at point *P*1 and *t_P_*_2_ is the timestamp of the NMEA at point *p*2.

**Figure 5:**
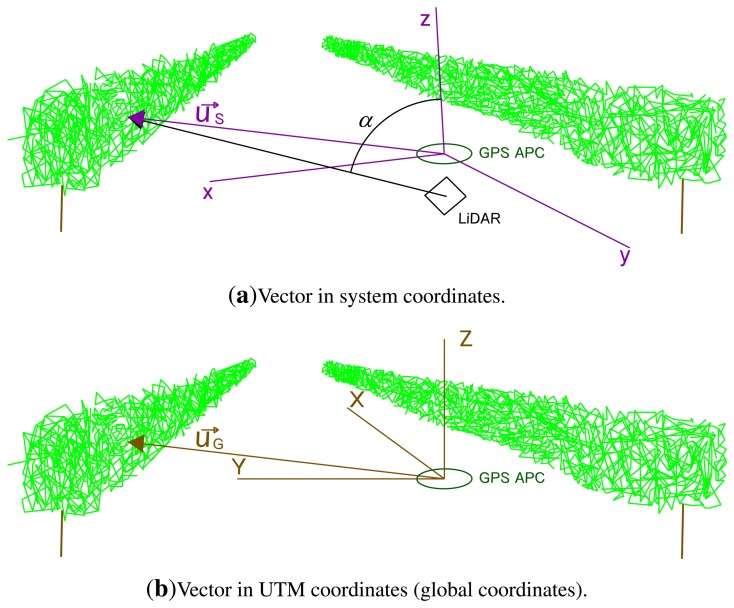
Vector coordinates (*x, y, z*) referring to the GPS APC (**a**) and the vector expressed in UTM coordinates (*X, Y, Z*) (**b**).

The second vector $\vec{{\text{u}}_{\text{G}}}$ (Figure 5b) corresponds to the intercepted point between the laser beam and the canopy in UTM coordinates. Calculating this vector requires the prior use of vector $\vec{{\text{u}}_{\text{S}}}$ (Figure 5a) that supplies the coordinates:

(4) $$\vec{{\text{u}}_{\text{S}}}=\left(\begin{array}{c} D_{\text{LiDAR}-V}\cdot \sin\left(\alpha \right)\\ 0\\ D_{\text{LiDAR}-V}\cdot \cos\left(\alpha \right) \end{array}\right)+\left(\begin{array}{c} x_{offset}\\ y_{offset}\\ z_{offset} \end{array}\right),$$

the coordinates of the intercepted point refer to the GPS APC in system coordinates. The parameters for computing this vector are the distance (*d_Li__dar−v_*) measured by LiDAR (Figure 2b), the zenith angle *α* (Figure 5a) of the laser beam and the offset from the GPS APC to the LiDAR sensor. These offsets are expressed in system coordinates (*x_offset_, y_offset_* and *z_offset_*). Then, the vector $\vec{{\text{u}}_{\text{S}}}$ is the position of the leaves with respect the GPS APC. To transform the vector $\vec{{\text{u}}_{\text{S}}}$ into UTM coordinates, $\vec{{\text{u}}_{\text{G}}}$, the azimuth of the path obtained from the GPS points, *Az*, and the inclination of the two axes obtained from the IMU, *ϕ* and *θ*, are necessary. Both corrections are possible using Equations (5)-(9), (11):

(5) $$\Delta X=X_{P2}-X_{P1}$$

(6) $$\Delta Y=Y_{P2}-Y_{P1}$$

(7) $$Az=\text{atan}2\left(\frac{\Delta X}{\Delta Y}\right)+\frac{\pi }{2}$$

(8) $$\phi =\text{atan}\left(\frac{R\left(3,2\right)}{R\left(3,3\right)}\right)$$

(9) θ=atan(R(3,2))

where *R*(*i, j*) are elements of the R matrix obtained from the IMU. A rotation matrix *R*_SG_ is obtained by introducing *Az, ϕ* and *θ* in Equation (2). Then, the $\vec{{\text{u}}_{\text{G}}}$ vector is:

(10) $$\vec{{\text{u}}_{\text{G}}}=R_{\text{SG}}\cdot \vec{{\text{u}}_{\text{S}}}$$

The absolute coordinates (UTM) of intercepted points are obtained by applying the expression:

(11) $$\left(\begin{array}{c} X_{\text{Point}}\\ Y_{\text{Point}}\\ Z_{\text{Point}} \end{array}\right)=\vec{{\text{u}}_{\text{G}}}+\vec{\text{v}}+\left(\begin{array}{c} X_{\text{P}1}\\ Y_{\text{P}1}\\ Z_{\text{P}1} \end{array}\right)$$

**Figure 6:**
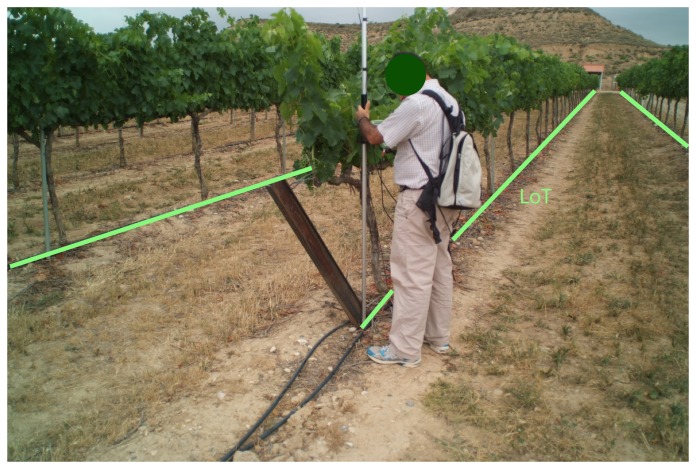
Process of measuring the line of trunks (LoT) with RTK-GPS.

**Figure 7:**
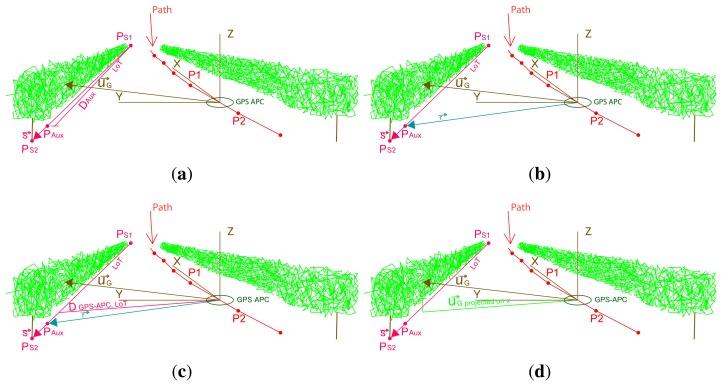
Parameters involved in filtering points that exceed the line of trunks (LoT). (**a**) Distance and auxiliary point; (**b**) the *r⃗* vector is far from the GPS APC to the auxiliary point *P*_Aux_; (**c**) the distance from the GPS APC to the LoT measured perpendicularly to the *s⃗*; (**d**) projected distance from the GPS APC to the vegetation, D_GPS APC-V_*x*__ .

Filtering was performed on a scan basis, discarding the notion of filtering the whole point cloud, because it would be more difficult and time consuming. Three geometric filters were programmed. The first filter removed points that did not intersect anything. By default, the LiDAR system assigns a maximum range distance (8.191 m) to these measurements, and they were all eliminated. The second filter deleted points impacting beyond the line of the trunks (LoT), *i.e.*, the axis of the vegetation wall. The LoT establishes a line to differentiate both sides of the vegetation wall. Since the system measured both sides of the row, our interest was to characterize the intercepted points for each half of the canopy, and it was necessary to establish a line that omitted from computation the points that cross over to the other half. To do this, the distance from the GPS APC to the line of trunks was first calculated. Then, if the horizontal distance from the intercepted point to the GPS APC was greater than the LoT, the point was omitted. In order to create the reference LoT, two points were measured, one at the beginning of the row, *P_S_*_1_, and the other, *P_S_*_2_, at the end, using topography procedures (Figure 6). Equations (13)–(16) allow us to locate an auxiliary point (*Paux*) (Figure 7a) in the LoT, near the position of the system.

(12) $$\vec{s}=P_{S2}-P_{S1}$$

(13) $$D_{\text{Axu}}=P_{\text{GPS APC}}-P_{S1}$$

(14) $$P_{\text{Axu}}=D_{\text{Axu}}\cdot \frac{\vec{s}}{\left|\vec{s}\right|}$$

In order to obtain *P*_aux_, it was necessary to calculate *D*_aux_, the distance from the first topographic point to the GPS APC sensor. Furthermore,

(15) $$\vec{r}=P_{\text{Axu}}-P_{\text{GPS APC}}$$

where *r⃗* (Figure 7b) is a vector from the GPS APC position to the auxiliary point *P*_aux_. In short, the desired distance can be known at each position using:

(16) $$D_{\text{GPS APC}‐\text{LoT}}=\frac{\left|\vec{r}\times \vec{s}\right|}{\left|\vec{s}\right|}$$

where *D*_GPS APC-LoT_ (Figure 7c) is the distance from the GPS APC to the LoT and is calculated by the vector product of the *r⃗* and *s⃗* vectors, divided by the norm of *s⃗*. To know if a canopy point was located farther than the LoT, the previous distance was compared with the norm of the x coordinate of the vector of the intercepted point:

(17) $$D_{{\text{GPS APC}‐\text{V}}_{x}}=\left|{\vec{u}}_{\text{G}}\left(1,1\right),{\vec{u}}_{\text{G}}\left(2,1\right),0\right|$$

where *D*_GPS APC-v_*_x_* (Figure 7d) is the distance from the GPS APC to the vegetation (the software only considers the horizontal plane and, therefore, only works with the x and y coordinates).

**Figure 8:**
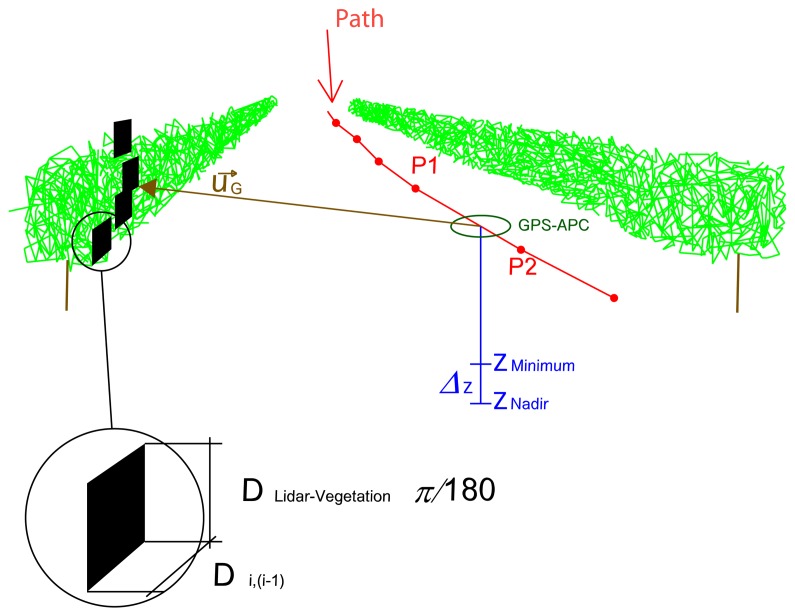
Minimum height to eliminate ground points and calculation of PLWA.

The third and final filter consisted of identifying the points located on the ground or points that are not part of the wall of vegetation. This filter established the nadir Z of the LiDAR sensor, plus an offset Δ*Z* considered as the ground height [5]. Any point that was below this *Z_Minimum_* was omitted from the computation (Figure 8). Specifically, the filter took the ground height in each scan, and Equation (18) was applied to set the minimum height:

(18) $$Z_{\text{Minimum}}=Z_{\text{Nadir}}+\Delta Z$$

After filtering, we are able to estimate the PLWA for each intercepted point or lateral projected area (*A_i_*). At each point of impact to the vegetation, we assigned the following area:

(19) Aij=Di,(i−1)⋅DLiDAR−V⋅π180

where $A_{i}^{j}$ is the area of point *j* within the scan *i, D_i_*_,(_*_i_*_−1)_ is the distance between the present scan *i* and the previous one, *i* − 1, *D_LiDaR__−V_* is the distance measured *by* LiDAR and $\frac{\pi }{180}$is the conversion factor from degrees (angular resolution of the LiDAR sensor) to radians (Figure 8). It is important to emphasize that these data points were distance, LoT and ground-filtered. On the other hand, the total projected area is:

(20) $$A_{i}=\sum_{i=1}^{n}A_{i}^{j}$$

where *A_i_* is the total projected area (or lateral area) assigned to scan *i* and *n* is the number of impacts on the vegetation for scan *i*. This value can be assigned to the 2D coordinates:

(21) $$\bar{X_{i}}=\sum_{i=1}^{n}\frac{X_{i}^{j}}{n}$$

(22) $$\bar{Y_{i}}=\sum_{i=1}^{n}\frac{Y_{i}^{j}}{n}$$

Thus, in these last steps, the system is transformed from a 3D system to a 2D system.

## 3. Results and Discussion

Four different experiments were conducted to validate the system. Each experiment worked on a different subsystem.

### 3.1. Synchronization of Sensors

**Figure 9:**
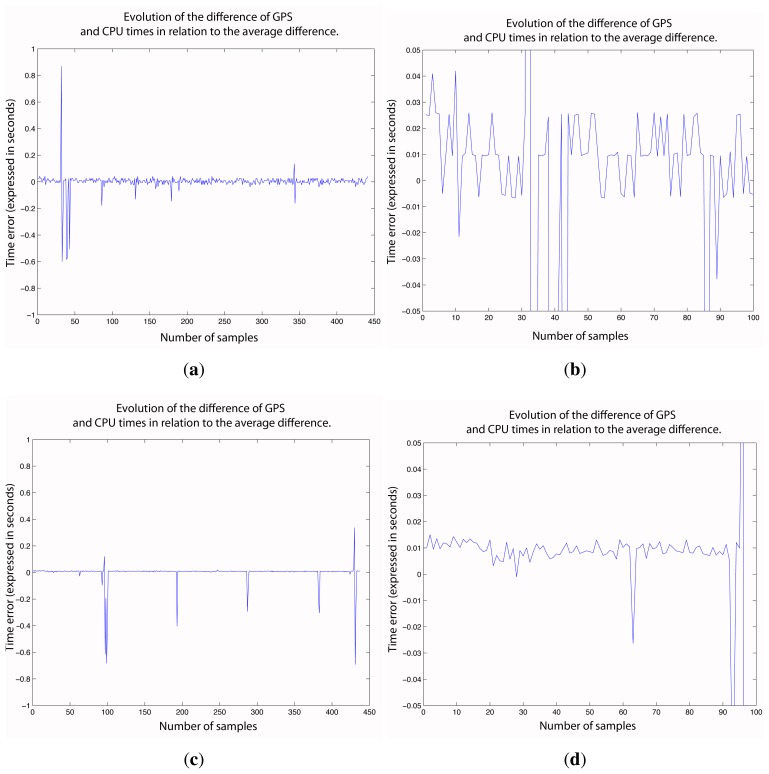
Synchronization errors in the operating systems. (**a**) Time error in Windows OS; (**b**) zoom on Figure 9a; (**c**) time error in Mac OS X, Snow Leopard; (**d**) zoom on Figure 9c.

As all of the information received by the CPU is timestamped with the CPU time and synchronized with the GPS time, it is very important that $\Delta t=t_{i}^{GPS}-t_{i}^{CPU}$ remains stable, because we use it to interpolate the time correction between two different GPS epochs. In order to validate this requirement, we calculated the average of the different GPS-CPU times, $\bar{\Delta t}$, and the deviation of every single measurement, $\delta \Delta_{t}=\Delta t-\bar{\Delta t}$, in a sequence of measurements. This test was carried out in Windows and in Mac OS X Snow Leopard. A vineyard row 420 m in length was used to check the synchronization. The final accuracy shown by the filter indicates the absolute precision, because it shows errors of positioning. Synchronization errors can be seen in Figure 9a, c.

Several findings can be drawn from the synchronization study. First, measurements obtained with the Mac OS had deviation jumps at certain times. However, with the Windows OS, such deviation jumps did not occur, which suggests that the problem originates from the Mac CPU. Other measurements showed the same tendency. More or less, 2% (percentage of the number of affected deviations with respect to the number of total deviations) of the measurements is affected by such phenomena.

However, it is also true that the Mac OS CPU time had a precision that was ten-times greater than the Windows CPU time. It appears that the Mac OS X manages CPU time with more precision than Windows OS.

In summary, synchronization problems result in errors of 0.02 s and 0.07 s using Mac and Windows, respectively. This involves positioning errors of up to 2 and 7 cm in each case, with a forward speed of 1 m/s.

### 3.2. Coordinates of the Intercepted Points Using LiDAR

Three different tests were carried out to analyze the relative accuracy of the resulting point cloud. In each one, two point clouds were compared, which is why this test showed relative accuracy. The first two tests consisted of measuring the distance from each point in the first point cloud to the closest point in the second point cloud or reference point cloud. As the point clouds could have different sizes, some points might not have a corresponding point in the reference point cloud.

**Figure 10:**
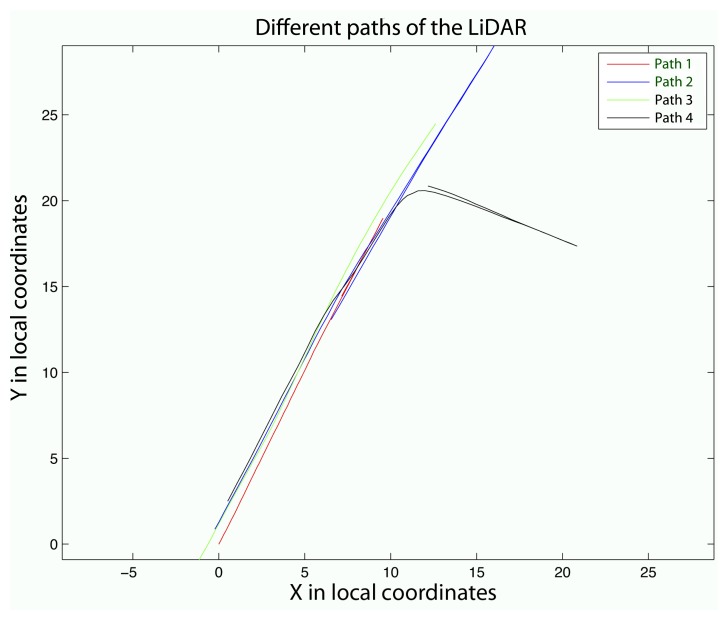
Comparison between the four paths.

The first geometry test consisted of measuring the same element in four different paths. The cloud of points obtained for Path 2 was used as the reference because the measurements took the whole element and had the simplest trajectory. It turned out that it was the path with the best precision. Distances between the points obtained for Paths 1, 3 and 4 and those closest to those obtained for Path 2 were computed. Figure 10 shows the different paths followed by the terrestrial laser system.

**Figure 11:**
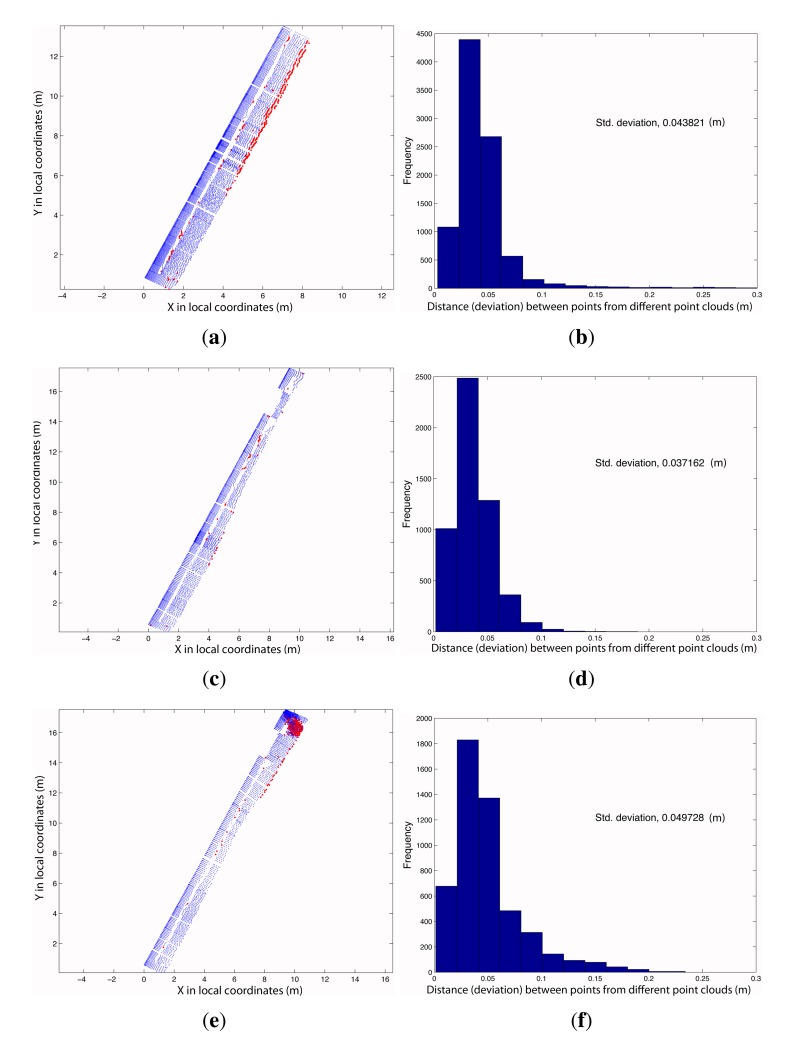
Comparison between four different point clouds produced by four different paths. Red points represent points with errors up to 10 cm. (**a**) Point Cloud 1, where points with errors greater than 0.10 m are represented in red; (**b**) histogram of distances (deviations) between point Clouds 1 and 2; (**c**) point Cloud 3, where points with errors greater than 0.10 m are shown in red; (**d**) histogram of distances (deviations) between point Clouds 3 and 2; (**e**) point Cloud 4, where points with errors greater than 0.10 m are shown in red; (**f**) histogram of distances (deviations) between point Clouds 4 and 2.

The reference path (Path 2) was characterized by performing a test for a path back and forth. Path 4 describes a turn on a round-trip path. However, Paths 1 and 3 were straight one-way paths. Figure 11 shows the results.

Red dots belonging to Clouds 1 and 3 are points that were located beyond point Cloud 2. In point Cloud 4, there was a concentration of points with errors at the corner of the element that was scanned. These point errors were due to the offsets or distances between the GPS APC and the LiDAR sensor that were not taken into consideration. This produces a coordinate translation. If the orientations of the system are similar in different paths, the differences between point clouds are small, and all of them have an error equal to the offset vector of LiDAR-GPS APC. If a large change in the orientation of the LiDAR were produced, this vector would be oriented in some other direction, and the resulting differences would be significant.

**Figure 12:**
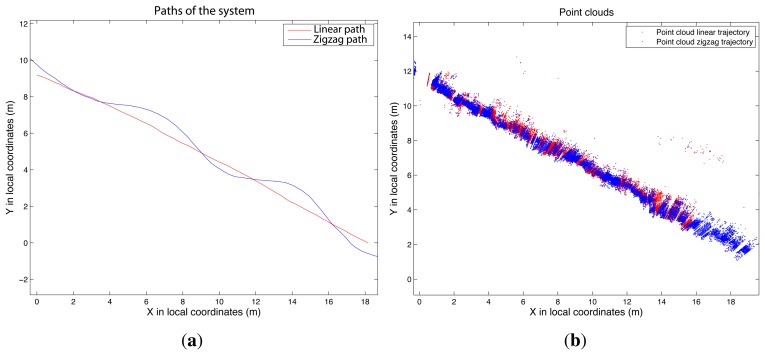
Zigzag path and linear path (**a**) and comparison between the respective point clouds (**b**).

Figure 12 shows the results of the second test. In this case, the LiDAR system was used in a vineyard to scan a length of 20 m on one side of a row. The novelty in this case is that the LiDAR sensor moved along two types of paths, straight and zigzag.

**Figure 13:**
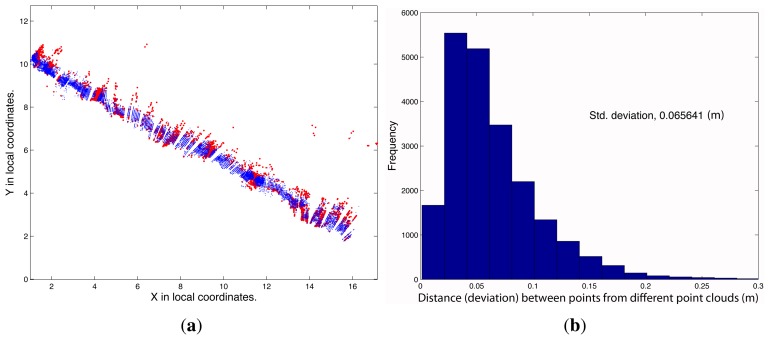
Comparison between linear path point cloud and zigzag path point cloud. (**a**) Zigzag point cloud and points with distances greater than 0.10 m (red points); (**b**) histogram of distances (deviations) between point clouds corresponding to linear and zigzag paths.

The first qualitative analysis leads to the conclusion that both point clouds show some consistency. However, it is difficult to compare the two point clouds, because the vineyard has a complex geometry. In fact, both point clouds were affected by errors (gaps in both point clouds), especially because the forward platform did not have a steering system with a turn capacity to follow the zigzag path. Then, sometimes, it was necessary to introduce a change of path direction by dragging. Comparing the distances from each point of the zigzag point cloud to the closest point in the linear path, it can be concluded that deviations were present. Figure 13a shows the deviations.

**Figure 14:**
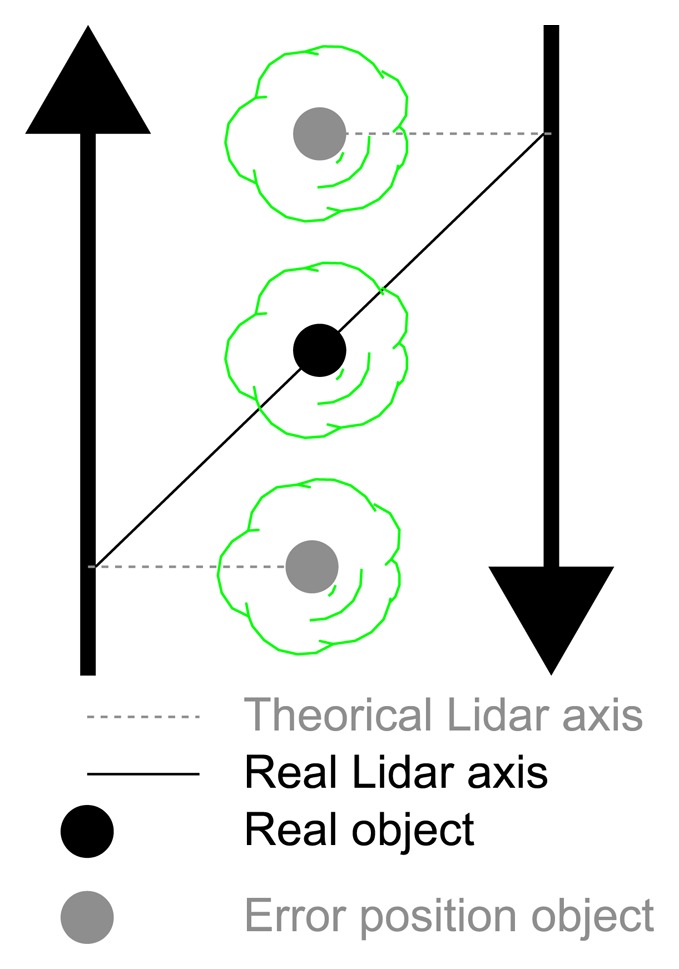
Error produced by misalignment of the LiDAR target axis.

**Figure 15:**
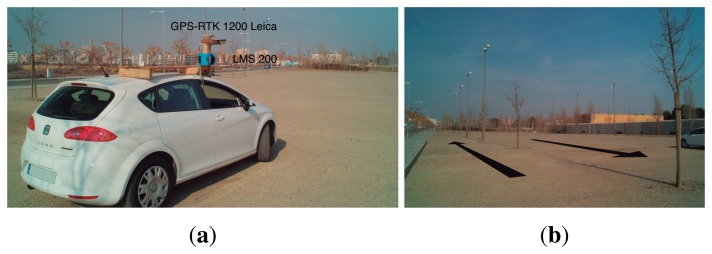
Setup of test: test for checking the perpendicularity of the scanning plane. (**a**) LiDAR mounted on a car; (**b**) test zone showing car paths.

The third test studied the coincidence of vertical linear elements in order to detect some of the systematic geometrical errors that can occur when the same element is measured from opposite directions. In this way, we tested whether the plane in which the LiDAR sensor works was perpendicular to the movement of the system. The same element would appear twice if there were any lack of perpendicularity (Figure 14). To do this, the GPS antenna and the LMS 200 were setup on top of a car, as shown in Figure 15a. The GPS receiver, computer and power supply were located inside the car. The car followed two different paths, shown in Figure 15b. Tree trunks and street lights of approximately 0.1 m and 0.2 m in diameter, respectively, were used to study the coordinate coherency. Passes were performed on both sides of the trees and lights, as seen in Figure 15b.

**Figure 16:**
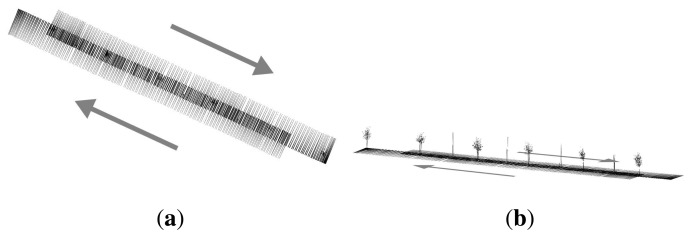
Test for assessing the perpendicularity of a laser beam pulsed by LiDAR. (**a**) Top view of resulting cloud; (**b**) axonometric view of resulting cloud.

**Figure 17:**
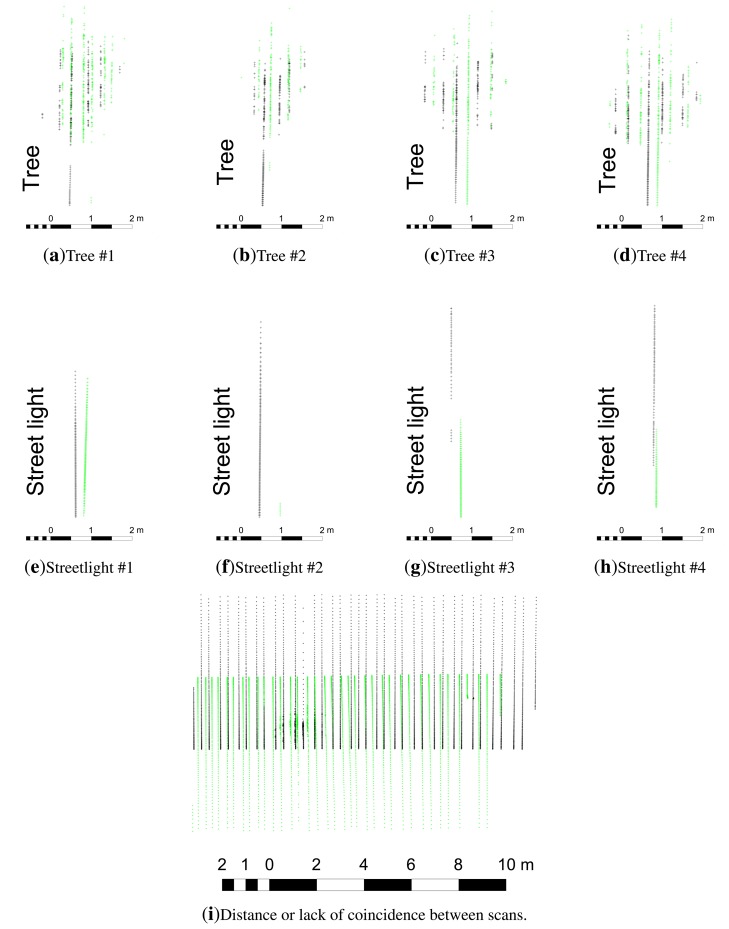
Tree and street light point clouds and distance between scans for the two paths. Graph scales are in meters. Black and green lines belong to forward and backward paths, respectively.

The system tested is shown in Figures 16–17i.

The results (Figure 16a,b) showed a certain coherency between the two point clouds. Nevertheless, the more detailed studies presented in Figure 17 showed errors of about 0.30 m. This difference was expected, given the low density of the scans in the area of study, the size of the objects and the lack of coincidence between the scans made in the opposite directions (Figure 17i).

### 3.3. Data Filtering

**Figure 18:**
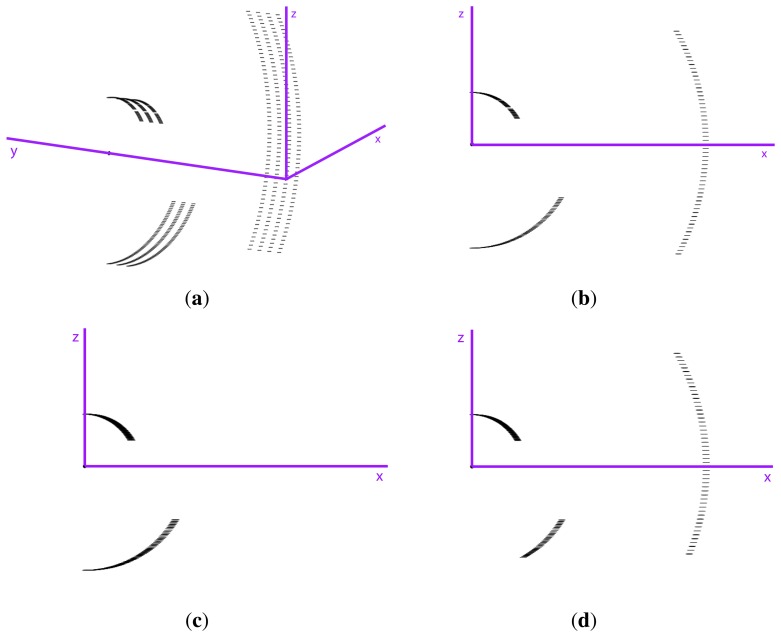
Filters to delete points not intercepted in the tree canopy. (**a**) Simulated point cloud in axonometric projection; (**b**) Simulated point cloud in front view; (**c**) Point cloud after applying distance filter; (**d**) Point cloud after applying ground filter.

**Figure 19:**
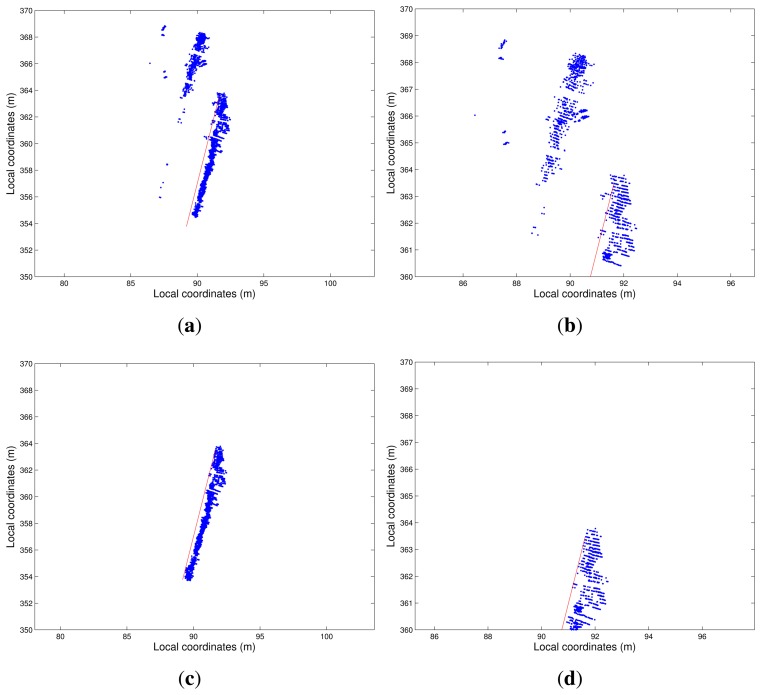
Result of the line filter application. The figures are shown in a birds-eye view. (**a**) Non-filtered points; (**b**) non-filtered points; (**c**) filtered points; (**d**) filtered points.

To check the goodness of the filtering process, a test was performed by running the programs with prior knowledge of which points had to be removed from the point cloud. The filters for maximum distance and ground points were tested with simulated data. We were able to program a simulated temporal sequence of LiDAR data. If all of the distances were equal, we would obtain a semi-cylinder. In the same way, if we introduce three different radii, we obtain three cylinder portions. These data provide a perfect scenario in which to test the filters, as we have precise knowledge of the point cloud. Regarding filtering points exceeding the LoT, the point cloud was drawn in two dimensions to verify that there were no measurements beyond the division line. The results of applying the filters are shown in Figures 18 and 19. All three filters worked correctly.

Figure 18a,b show the results of simulating three complete scans of 180^°^. Subsequent filtering of the data (distance and ground points) leads to the results shown in Figure 18c,d. Figure 18c shows that more distant points have been eliminated. On the other hand, points under a fictitious Z value have also been removed in Figure 18d.

The results of the line filter can be appreciated in the images presented in Figure 19. The point cloud shown in this figure has been taken from one side of the row. Figure 19a,b correspond to the point cloud without line filtering, while Figure 19c,d show the result after filtering. If we contrast the filtered point cloud and the original unfiltered point cloud, we can see that the points beyond the line have been deleted.

### 3.4. Usefulness of PLWA to Estimate the Leaf Area Index

**Figure 20:**
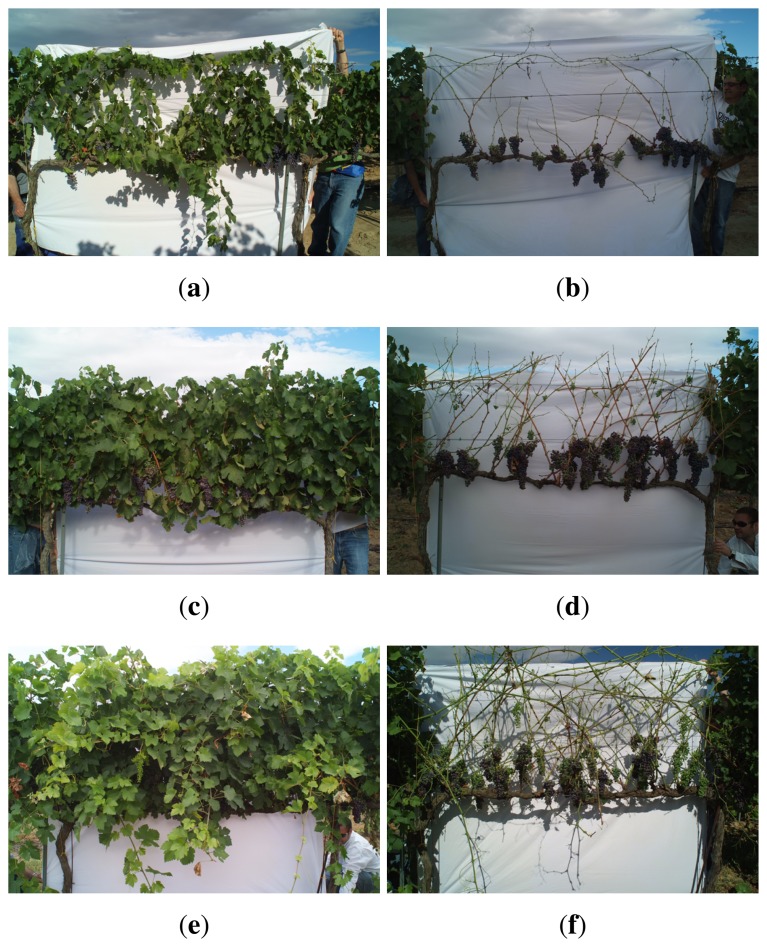
Vineyard row sections with different levels of vigor. (**a**) Row section of low vigor; (**b**) row section of low vigor after defoliation; (**c**) row section of medium vigor; (**d**) row section of medium vigor after defoliation; (**e**) row section of high vigor; (**f**) row section of high vigor after defoliation.

The proposed LiDAR system allows us to obtain the PLWA. This system improves the measurement of LWA, since it is an objective system (*i.e.*, independent of user action) capable of sampling targets separated by a few centimeters, overcoming the homogenization and subjectivity of manual measurements. To study the possible relationship between the PLWA and certain vegetative parameters of interest, a test was designed to relate the PLWA and the Leaf Area Index (LAI). Three vineyard row sections 2 m in length were selected within the zone of measurements, with the aim of representing three different levels of vigor: low, medium and high (Figure 20a,c,e). Each section was then scanned by LiDAR on both sides of the row, and later, vines were manually defoliated to obtain the actual value of the leaf area index (Figure 20b,d,f). After applying the LiDAR system, the three blocks were defoliated to measure real leaf area using an Area Measurement System-Conveyor Belt Unit; Delta-T Devices Ltd, Cambridge, UK.

**Figure 21:**
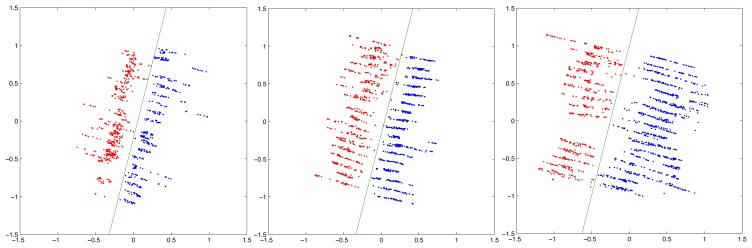
Top view of the point clouds for the different row sections, low vigor (**left**); medium vigor (**center**); and high vigor (**right**). The gap on the left side in the third section was due to some problem in the data acquisition. Local coordinates were used to plot the points (m).

Figure 20 shows the point cloud for each of the row sections. The most interesting feature is the distinction between the points on the right and left sides of the row. In general, vines with greater vigor (high LAI value) also show higher canopy width. However, the canopy is not always symmetrical (at least in vines with more vegetation, Figure 21), and hypothetically, one would expect a greater pixelated leaf wall area on those sides of the row with a greater half-canopy width.

**Figure 22:**
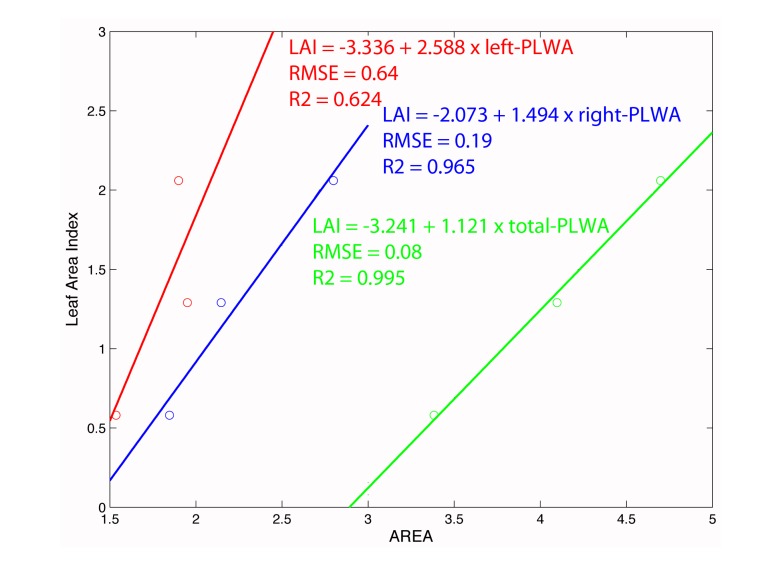
Linear regression between LAI and PLWA.

Table 1 shows the results of this analysis. Increasing vigor (high LAI values) also increases the value of the PLWA, and this is true in all cases, except for the leaf wall area obtained when more vigorous vines were scanned from the left side. However, this result was expected, given the lack of data (gap) produced when this side was scanned. On the other hand, the hypothesis of obtaining a higher value of Leaf Area Index with higher PLWA values seems reasonable in that a greater amount of vegetation is often associated with a greater width of the half-canopy, which increases the likelihood of intercepting leaves with LiDAR and obtaining a higher PLWA value. However, using the PLWA from only one side of the row to estimate the LAI for the entire width of the canopy could lead to questionable results. Estimating the total leaf wall area (right and left sides) is probably the best option, Figure 22.

Three aspects are noteworthy from the above analysis. First, results from the regression models are conceptually consistent with the LiDAR operation in that the negative intercept is not a model error. Indeed, when LiDAR is applied to leafless vines, one can expect to obtain a vegetated area, as the laser beam can impact bunches of grapes and woody plant structure. Second, LAI estimation is improved when the total PLWA (left and right) is used. However, the difficulty of obtaining this parameter in real time raises the need to use the lateral PLWA to estimate the LAI corresponding to the half-canopy, *i.e.*, the amount of vegetation that lies between the line of the trunks and the outer face of the canopy. Finally, the PLWA does not necessarily coincide with the LWA. The fundamental difference is that the proposed system takes into account the porosity of the canopy. Holes in the canopy are not accounted for as effective area. Moreover, the system yields different PLWA values depending on which side of the row (right or left) the LiDAR has been applied. These differences may be higher for asymmetric vines.

## 4. Conclusions

The developed terrestrial LiDAR system allows lateral scanning of vegetation in tree crops. The LiDAR system is further equipped with an RTK-GPS receiver for georeferencing point clouds and an inertial sensor to estimate the inclination of the system during the measurements.

A validation is performed for each of the component subsystems. The synchronization subsystem achieved a precision of 0.07 s in the worst conditions. Therefore, at the usual speed of 1 m/s, the error would be 0.07 m.

In contrast, the precision of the point cloud is consistently less than 0.1 m, even when the LiDAR sensor is moved along the alleyways delineating irregular paths.

Finally, the promising results reported here in predicting the LAI from the PLWA also confirm the reliability of the system in terms of data acquisition and processing.

## Figures and Tables

**Table 1:** Leaf area index (LAI) and pixelated leaf wall area (PLWA).

**Vigor**	**Actual LAI**	**Right PLWA**	**Left PLWA**	**Total PLWA**
Low	0.58	1.8467	1.5364	3.3831
Medium	1.29	2.1463	1.9508	4.0971
High	2.06	2.7986	1.8998	4.6984
